# Update on Minimally Invasive Glaucoma Surgery (MIGS) and New Implants

**DOI:** 10.1155/2013/705915

**Published:** 2013-11-27

**Authors:** Lívia M. Brandão, Matthias C. Grieshaber

**Affiliations:** Department of Ophthalmology, Glaucoma Service, University Hospital of Basel, University of Basel, Switzerland

## Abstract

Traditional glaucoma surgery has been challenged by the advent of innovative techniques and new implants in the past few years. There is an increasing demand for safer glaucoma surgery offering patients a timely surgical solution in reducing intraocular pressure (IOP) and improving their quality of life. The new procedures and devices aim to lower IOP with a higher safety profile than fistulating surgery (trabeculectomy/drainage tubes) and are collectively termed “minimally invasive glaucoma surgery (MIGS).” The main advantage of MIGS is that they are nonpenetrating and/or bleb-independent procedures, thus avoiding the major complications of fistulating surgery related to blebs and hypotony. In this review, the clinical results of the latest techniques and devices are presented by their approach, ab interno (trabeculotomy, excimer laser trabeculotomy, trabecular microbypass, suprachoroidal shunt, and intracanalicular scaffold) and ab externo (canaloplasty, Stegmann Canal Expander, suprachoroidal Gold microshunt). The drawback of MIGS is that some of these procedures produce a limited IOP reduction compared to trabeculectomy. Currently, MIGS is performed in glaucoma patients with early to moderate disease and preferably in combination with cataract surgery.

## 1. Introduction

The common goal of glaucoma treatment is to lower intraocular pressure (IOP) to a level which is safe for the optic nerve head of an individual patient. Surgical treatment is usually required when topical medication and/or laser procedures are not tolerated and/or do not sufficiently reduce IOP. Trabeculectomy is still regarded as the “gold standard” in glaucoma surgery; however, less invasive procedures, so-called minimally invasive glaucoma surgery (MIGS), have gained interest and popularity among patients and surgeons over the past years because they claim to be safe and easily combined with cataract surgery. This article reviews the latest techniques and developments of MIGS which are classified here by their surgical approach, that is, ab interno and ab externo (Table 1).

## 2. Ab Interno Procedures

Ab interno procedures are performed under gonioscopic view usually through a small side port incision. The techniques can be divided by their way of principle, that is, by removing tissue or by implanting a shunt device.

### 2.1. Ab Interno Trabeculotomy (Trabectome)

**Figure 1:**
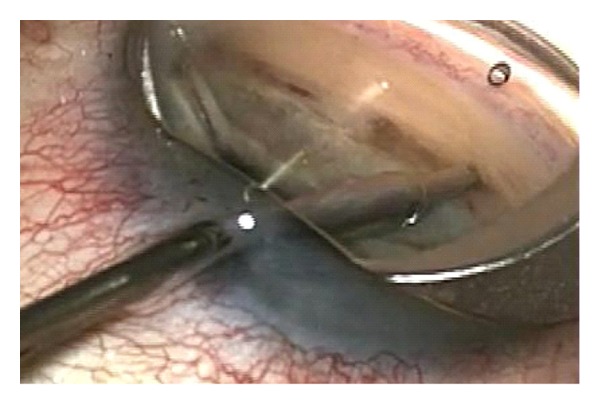
Ab interno trabeculotomy with the Trabectome device.

**Figure 2:**
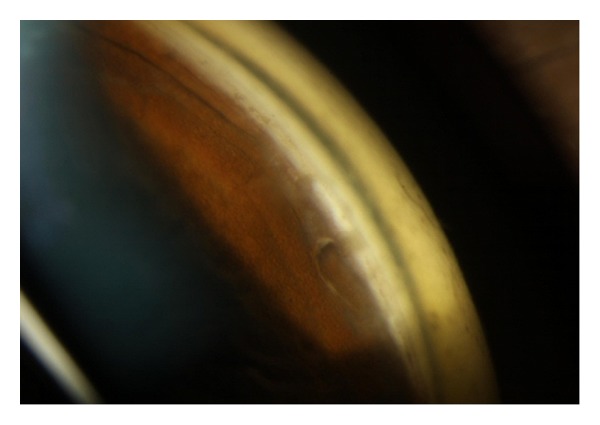
Postsurgery gonioscopic view of ab interno trabeculotomy.

Ab interno trabeculotomy by the Trabectome device (NeoMedix, Tustin, USA) uses a high frequency electrocautery to ablate the trabecular meshwork (TM) and inner wall of Schlemm's canal (SC). It consists of a disposable hand piece connected to a console with irrigation and aspiration controlled by a foot pedal with stepwise activation. The procedure is done under gonioscopic view (Figures 1 and 2). Depending on the surgeon decision, tissue of up to 90 or 120 circumferential degrees can be removed. The potential advantages for this angle surgery are the removal of the area of greatest resistance to aqueous outflow and tissue debris which may reduce the inflammatory stimuli and consequently potential scarring. Francis et al. [1] analyzed the histological effects of the application of different power levels in postmortem eyes. They reported that heat damage was not observed in deeper parts to the TM or surrounding tissues in all specimens analyzed.

In 2005, Minckler et al. [2] published the first pilot study with 37 patients with primary open-angle glaucoma (POAG), pseudoexfoliation glaucoma, pigment dispersion, and possible steroid induced glaucoma. Mean IOP dropped from 28.2 ± 4.4 mmHg preoperatively to 17.4 ± 3.5 at 6 months and mean number of medications from 1.2 ± 0.6 to 0.4 ± 0.6. All 37 patients presented blood reflux at the end of surgery, and 22 patients (59%) had hyphema on the first day after surgery. No serious complications occurred, but peripheral anterior synechiae, transient endothelial, or Descemet's membrane (DM) injuries and IOP spikes may be observed.

Since this first report of Minckler, the instrument supplier requires surgeons to provide demographic information and pre-, intra-, and postsurgical data on the first 20 patients at regular intervals. All the information collected from these surgeons is stored in a database, which serves as a growing source of data on the Trabectome surgical system and studies published below.

In a prospective interventional case series [6], 304 patients with open-angle glaucoma underwent combined trabeculotomy with cataract surgery. Mean preoperative IOP was 20.0 ± 6.3 mmHg, and mean postoperative IOP was 15.5 ± 2.9 mmHg, with a 1.4 ± 1.3 mean number of glaucoma medications after one year of follow-up. Nine patients needed additional glaucoma procedures. The great limitation of the analysis was the high number of losses during the 21 months follow-up.

Another large ongoing case series examined the outcomes of Trabectome alone versus combined procedures with phacoemulsification based on data from 1127 surgeries performed at 46 study sites since January 2006 [3]. At 24 months, IOP dropped by 40% from 25.7 ± 7.7 mmHg preoperatively to 16.6 ± 4.0 mmHg in the Trabectome alone group compared to 30% from 20.0 ± 6.2 mmHg to 14.9 ± 3.1 mmHg in the combined phaco-Trabectome group. Mean number of medications decreased from 2.9 to 1.2 in the Trabectome group and from 2.6 to 1.5 in the combined group. A total of 14% (100 patients) were considered failure cases from Trabectome alone group. Patients that needed subsequent trabeculectomy accounted for 5.9% of the total group.

In a prospective nonrandomized cohort analysis with 6 months follow-up [4], 1401 patients undergoing Trabectome procedures were grouped by baseline IOP levels. In the group with preoperative IOP levels ≤17 mmHg, the IOP mean reduction was as little as 7% mmHg with a 35% reduction in antiglaucomatous medications. However, in the group with preoperative IOP ≥ 30 mmHg, IOP reduction was as great as 48% with 25% reduction in antiglaucomatous medications.

According to a report from Ting et al. [39], exfoliative glaucoma patients may have, overall, a greater IOP reduction compared to POAG patients. After trabeculotomy, IOP decreased by 12.3 ± 8.0 mmHg in the exfoliation group and by 7.5 ± 7.4 mmHg in the POAG group. The IOP decrease was also greater in combined procedures with 7.2 ± 7.7 mmHg in exfoliation group and 4.1 ± 4.6 mmHg in POAG group.

Very recently, Maeda et al. published original data of 80 eyes of 69 Japanese patients [9] with a 6 months follow-up. They reported a drop from mean preoperative IOP of 26.6 ± 8.1 mmHg to 17.4 ± 3.4 mmHg after surgery. The number of antiglaucomatous medications lowered from 4.0 ± 1.4 to 2.3 ± 1.2 at 6 months. The patient population was selected here prior to the procedure which is different from all the other study reports which analyzed the results retrospectively from the Trabectome database; patients presenting corneal edema or opacities, vision of hand movements, shallow anterior chamber, uveitis, neovascularization, and difficulty for TM/scleral spur identification were excluded. No serious complications were reported. Thirteen patients needed further surgical intervention.

From all studies, most complications related to Trabectome were IOP spikes at first day and intraoperative blood reflux from SC with subsequent hyphema (Table 2). A recent case series reported a delayed-onset hyphema with vision disturbance 8.6 months on average after the procedure (range from 2 to 31 months) [40]. Such phenomenon is overall rare (12 out of 262 cases; 4.6%). Suggested triggers for delayed-onset hyphema may be the Valsalva maneuver, use of aspirin and warfarin, and an IOP below episcleral venous pressure with a physiologic blood reflux into the anterior chamber as the TM is removed and SC exposed after surgery.

Other typical complications of Trabectome trabeculotomy are goniosynechiae and membrane growth which may both lead to IOP elevation. According to Wang and Harasymowycz [41], Nd:YAG laser is useful to rupture the occluded areas and adhesions in order to restore the outflow pathway and to lower effectively IOP.

Regarding the complications, Jea et al. [42] reviewed medical records from patients who underwent (secondary) trabeculectomy after a failed trabectome procedure (group 1) compared to patients that underwent primary trabeculectomy (group 2). After a mean follow-up of 15.4 ± 9.8 months (group 1) and 18.6 ± 10.4 months (group 2), both groups showed no statistical significant difference in IOP over time. In the first group, IOP dropped from 27.6 ± 11.8 mmHg at baseline to 10.6 ± 2.6 mmHg (47.1%) and in group 2 from 29.2 ± 11.4 mmHg to 11.0 ± 5.4 mmHg (52.1%) at 24 months. The authors concluded that the Trabectome procedure did not influence the outcome when a subsequent trabeculectomy was needed.

Overall, Trabectome trabeculotomy achieved fairly good IOP levels ranging from 13.5 to 17.9 mmHg (Table 3). It is to note that the higher the IOP was before surgery, the greater the IOP reduction. From a pathophysiological perspective, it is remarkable that IOP levels are not lower since up to 1/3 of the total TM is removed. Possible reasons are late closure due to inflammatory and wound healing process and the resistance of the natural outflow pathway including episcleral venous pressure and sclera. The greatest limitation of the available literature on the Trabectome today is the bias in patient's selection, data collection, follow-up criteria, and the fact that almost all data analyses are based exclusively on the supplier's collected database.

### 2.2. Excimer Laser Trabeculotomy

**Figure 3:**
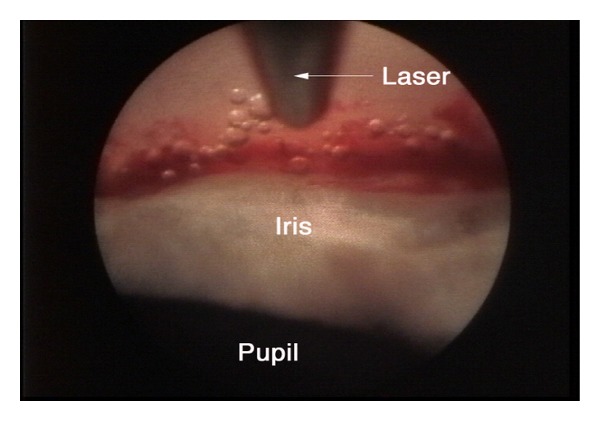
Refluxed blood and bubble formation represent successful photoablation (courtesy of J. Funk, MD, PhD).

Ab interno excimer laser trabeculotomy (ELT) (Glautec AG, Nurnberg, Germany) utilizes the energy of a xenon chloride pulsed excimer laser connected to a quartz fiber optic probe. The procedure intends to enhance outflow facility by creating microperforations in the TM and inner wall of SC. The probe tip is beveled at 65 degrees to aid the placement against the angle via gonioscopic or endoscopic guidance (Figure 3). Eight to ten laser punctures are spaced over 90 degrees, each pulse delivering a mean energy of 1.2 mJ over 80 ns duration [8]. Presence of blood reflux following the laser ablation means that SC has been accessed.

To date, there are two study groups reporting their experience with ELT. Babighian et al. [10] demonstrated an IOP-lowering effect from 24.8 ± 2.0 to 16.9 ± 2.1 mmHg in POAG refractory to topical medication after a mean follow-up of 2 years. Overall, 90.5% of the patients had an IOP reduction of 20% or more compared to baseline. Eight patients (38.1%) required additional medical therapy to achieve IOP control, and 9.5% failed the treatment. In a subsequent study, they compared ELT with 180 degrees selective laser trabeculoplasty (SLT) in the same study population [11]. They found no statistical significant differences in complete or qualified success rates between the two groups at 24 months although the percentage of IOP reduction was slightly higher in the SLT than in the ELT group (29.6% versus 21%).

The other study group of Funk and colleagues compared results from open-angle glaucoma (OAG) patients treated with ELT alone (75 eyes) versus ELT combined with phacoemulsification (60 eyes) [12]. Patients treated only with ELT achieved an IOP reduction of 30% (24.1 ± 0.7 mmHg to 16.8 ± 1.0 mmHg), while patients treated with the combined procedure had a decrease in IOP of 47% (22.4 ± 0.6 mmHg to 12.8 ± 1.5 mmHg) at last follow-up. Antiglaucomatous drugs dropped from 1.9 ± 0.1 to 1.5 ± 0.3 in the ELT alone group but paradoxically increased from 1.1 ± 0.2 to 1.8 ± 0.9 in the combined group. In a second study, the same group evaluated the effect of ELT combined with phacoemulsification in patients with POAG and secondary OAG [13]. They found an average IOP reduction of 34.7% (0.79 mmHg ± 1.50, *P* < 0.001) after one year follow-up.

Present data suggest that ELT lowers IOP in a selected subgroup of patients, if the procedure is combined with phacoemulsification (Table 4). The technique has been described as simple and quick and requires only topical anesthesia. Reported advantages are a low incidence of complications and no interference with future fistulating surgery if needed. A slight bleeding is expected during the procedure and may even be interpreted as patency of the TM with a good prognosis regarding IOP reduction. However, IOP increase in the first 24 hours and fibrin reaction may also be seen following ELT. Further, ELT requires expensive equipment and experience from the surgeon working with a direct-view goniolens.

### 2.3. Trabecular Microbypass Stent

**Figure 4:**
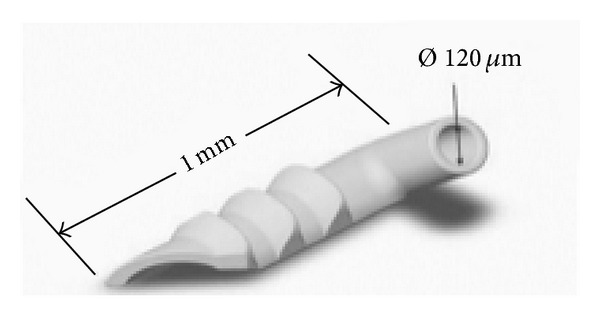
Gonioscopic view of implanted transtrabecular microbypass (iStent) in situ (courtesy of Glaukos Corporation).

**Figure 5:**
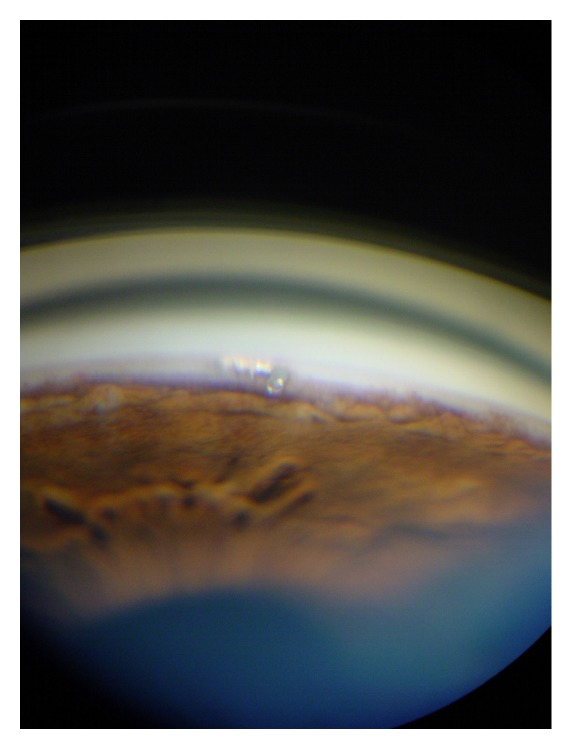
Microbypass design and dimensions (courtesy of Glaukos Corporation).

The trabecular micro-bypass stent (iStent, Glaukos, Laguna Hills, CA, USA) was designed to create a permanent communication between anterior chamber and SC overcoming the primary site of increased outflow resistance. It is a 1.0 mm long single piece heparin-coated nonferromagnetic titanium device with three retention arches in its outer surface to ensure secure placement. The device is implanted with the use of a disposable insertion instrument by an ab interno gonioscopic guided approach (Figures 4 and 5).

Numerous studies have evaluated the efficacy and safety of the microbypass alone or combined with phacoemulsification (Table 5). Most of them included a limited number of patients and had a follow-up time of one year or less. Among the listed studies, the mean IOP reduction was 5.3 mmHg with a decrease percentage ranging from 16.3% to 31%. Mean reduction in the number of medications was 1.2 (range 0.47 to 2.0).

A large prospective, randomized, open-label, controlled, and multicenter clinical trial was recently published by Samuelson et al. [20]. They included 240 eyes of patients with mild to moderate OAG with a controlled IOP on 1 to 3 drugs undergoing cataract surgery alone or combined with stent implantation. The follow-up period was up to 12 months. At 1 year, 72% of eyes in the combined treatment group and 50% of eyes in the cataract group achieved an IOP 21 mmHg or less with no additional medication (*P* < 0.001). Mean IOP achieved was 17.0 ± 2.8 mmHg in the combined group and 17.0 ± 3.1 in the cataract group. Although the reduction in IOP was more than 30% in both groups, the number of medications required to maintain a similar IOP level was greater in the control group at 12 months (*P* < 0.005). In the two-year follow-up report of the same study group, Craven et al. [22] confirmed the stability of IOP over time with a mean of 17.1 ± 2.9 mmHg at 24 months in the combined group and a mean IOP of 17.8 ± 3.3 mmHg in the cataract group. The number of ocular hypotensive medication to reach an IOP of 21 mmHg or less was slightly lower in the combined group compared to the cataract group.

In a long-term study on 19 patients undergoing combined phacoemulsification and micro-bypass implantation [21], the mean IOP decreased from 19.4 ± 1.9 mmHg to 16.3 ± 4.2 mmHg at 53.7 ± 9.3 months. The number of hypotensive medication dropped from 1.3 ± 0.5 preoperatively to 0.8 ± 0.9 postoperatively. Eight patients (42%) achieved satisfactory IOP with no additional medications at the end of follow-up. The authors stated that at 5 years IOP was significantly lower than preoperatively, although patients required a similar mean number of antiglaucomatous medications to achieve target pressure after surgery.

To date, the majority of the micro-bypass studies have shown only a small to moderate IOP reduction with the implantation of one stent, and so inserting more than one stent may further lower IOP. This was the aim of the study of Fernández-Barrientos et al. [18]. They used fluorophotometry to investigate the impact of multiple stents implantation on aqueous humor outflow in vivo. Patients included in the study had indication for cataract surgery and concomitant OAG or ocular hypertension. A group of 33 patients were randomized to receive phacoemulsification alone or combined with stent implant (two stents). Aqueous flow and trabecular outflow facility were calculated before and at specific time points after surgery. Aqueous outflow augmented from 1.78 ± 0.44 *μ*L/min preoperatively to 2.94 ±1.52 *μ*L/min (*P* = 0.040) at 12 months in the stent group and from 1.74 ± 0.82 *μ*L/min to 2.12 ± 0.74 *μ*L/min (*P* = 0.546) in the control group. Trabecular outflow facility also showed an increase from baseline of 0.32 ± 0.16 *μ*L/min/mmHg in the stent group and 0.05 ± 0.08 *μ*L/min/mmHg in the control group (*P* = 0.02). Although the difference between IOP values at 12 months demonstrated a borderline statistical significance (*P* = 0.04), the difference in the number of medications in each group was significant at 12 months (*P* = 0.007), suggesting that the implantation of the trabecular bypass lowers the number of additional medications (0.0 drugs in stent group and 0.7 ± 1.0 in control group).

In a nonrandomized prospective case series, Belovay et al. [23] compared IOP among patients with 2 or 3 stents implanted. Both groups had combined procedures with cataract surgery. The authors included not only patients with POAG and pseudoexfoliative glaucoma but also patients with a so-called mixed mechanism characterized by a history of angle closure glaucoma and open angles after iridotomy at the time of surgery. In the 2 stent group (28 patients), mean IOP lowered from 17.3 mmHg to 13.8 mmHg (*P* < 0.001) with a reduction in the mean number of medications from 2.8 to 1.0. In the three stent group (25 patients), mean IOP decreased from 18.6 mmHg to 14.8 mmHg (*P* < 0.001), with a reduction in the mean number of medications from 2.6 to 0.4. Overall 70% (37 eyes) had an IOP of 15 mmHg or less at 12 months. Considering the different mean IOP reduction with variable numbers of stents, the surgeon may determine the numbers of stents implanted according to target IOP of each patient (individual titration).

Today, a second and third generation of the original stent have been developed. In a study on enucleated eyes, Bahler et al. [43] assessed the morphology of TM after implantation of a second generation stent (iStent inject) by scanning electron microscopy and 3-dimensional microcomputed tomography. The authors observed an increased and sustained outflow facility from 0.16 ± 0.05 *μ*L/min/mmHg to 0.38 ± 0.23 *μ*L/min/mmHg (*P* < 0.03) and a further increased outflow after second implant in the perfused eyes segments. At present, there are 6 ongoing studies with the second and third generation devices, but no clinical outcomes data are yet published.

Regarding complications (Table 6), the most common reported adverse events with the trabecular micro-bypass are the following: mild hyphema, transient IOP spike, corneal edema, stent obstruction, anterior chamber collapse, inability to implant the stent, vitreous incarceration, stent malpositioning, and need of secondary surgery. Obstruction of the stent lumen by blood clot or iris may resolve spontaneously or following iridoplasty.

There is only one recent case series that focused on the safety and efficacy of the stent alone implantation [15]. In a small group of 10 patients with secondary OAG, IOP was reduced from 26.5 ± 7.9 mmHg before surgery to 17.0 ± 2.5 mmHg after a mean follow-up of 12.7 ± 4.6 months. Two patients (20%) were excluded during the study. The average number of hypotensive medication used preoperative was 2.9 ± 0.7 and showed a reduction of 1.1 ± 0.6 at 12 months (*P* < 0.05). Only one patient in the group achieved IOP of 18 mmHg or less without additional medication. These results should be interpreted with caution due to the small number of patients.

The general advantages of ab interno procedures were mentioned before. The specific advantage of the stent is theoretically the maintenance of the bypass patency by the heparin coat, although the created communication is limited by the small size of the stent's lumen. Combined procedures seem to achieve better results than stent-alone implantation [19]. The trabecular micro-bypass (iStent) was approved by the FDA and has the CE Mark from European Community as well. According to FDA recommendation the device is indicated for the use in patients with mild to moderate POAG associated with phacoemulsification.

### 2.4. Schlemm's Canal Scaffold (Hydrus)

**Figure 6:**
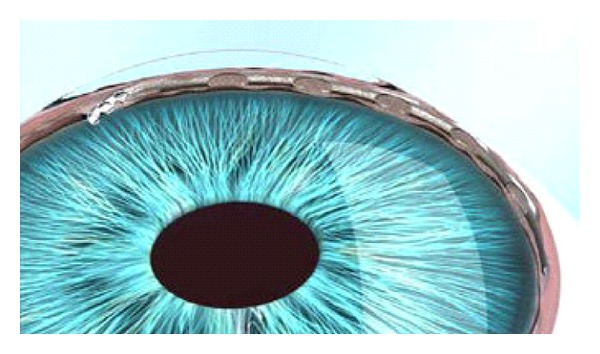
Hydrus scaffold design and positioning—illustration. (http://www.revophth.com).

The so-called “intracanalicular scaffold” (Ivantis, Inc., Irvine, CA, USA) is an 8 mm long device made of a highly elastic biocompatible material called nitinol, which has already been used in other implantable medical devices. The scaffold is placed inside SC during cataract surgery (Figure 6).

The idea of the Hydrus scaffold is to increase aqueous outflow from the anterior chamber to SC. A recent study on human cadaver eye segments determined the changes in outflow facility after implantation of the Hydrus microstent under different conditions of perfusion pressure and found that the Hydrus microstent increased outflow facility significantly compared to controls [44]. The same study group was also able to demonstrate with a modified version of the microstent an increase in outflow facility from 0.33 ± 0.17 *μ*L/min/mmHg to 0.52 ± 0.19 *μ*L/min/mmHg and a decrease in resistance from 4.38 ± 3.03 mmHG/*μ*L/min to 2.34 ± 1.04 mmHg/*µ*L/min. [45].

In a 2012 review, Saheb and Ahmed [46] cited unpublished data of 28 eyes with mild POAG undergoing phacoemulsification and implantation of the canal scaffold. Baseline IOP from 29.9 ± 5.8 mmHg dropped to 15.3 ± 2.3 mmHg at 6 months. Adverse effects included subconjunctival hemorrhage, hyphema, and peripheral anterior synechiae. The Hydrus scaffold device is available only for investigational use in the United States.

### 2.5. Suprachoroidal Microstent (CyPass)

The CyPass is a supraciliary tube designed to create a controlled outflow from the anterior chamber to the suprachoroidal space. It is made from polyamide and has a length of 6.35 mm and a diameter of largest 0.51 mm (Transcend Medical, Menlo Park, CA). Currently, the device is for investigational use only, and three clinical trials are registered.

A first interim report of an ongoing multicenter study has recently been published [47]. The trial included cataract patients with uncontrolled (cohort 1) and controlled (cohort 2) primary or secondary OAG. Patients underwent phacoemulsification followed by microstent implantation with at least 6 months of follow-up. The total cohort (184 patients) had a mean preoperative IOP of 21.1 ± 5.91 mmHg (before washout) and baseline number of antiglaucomatous medications of 2.1 (SD ± 1.12). At 6 months, cohort 1 had a 36.9% drop in IOP (*P* < 0.001) and mean number of medications of 0.9 ± 0.15, whereas cohort 2 had only a mean IOP decrease of 1.2 mmHg and final IOP of 15.6 ± 0.68 mmHg. As cohort 2 was medically controlled, the authors stressed that the important outcome was the reduction in the mean number of medications of 71.4% (0.6 ± 0.07). Among the adverse events, hypotony within the first month (13.8%) was the most common, followed by transient IOP increase (10.5%), inflammation of anterior chamber 4.4%, postoperative hyphema (1.1%), and others with incidence of less than 1%. Nine patients (5%) needed additional surgical intervention, but none of 184 patients needed stent removal.

### 2.6. Subconjunctival Implant (Aquesys)

This implant consists of a soft collagen tube with an inner diameter of 65 microns. It is placed via the anterior chamber (ab interno) into the subconjunctival space (AqueSys Inc.). The idea is to create subconjunctival filtration (bleb formation) without opening the conjunctiva. However, bleb-related problems are not solved with this procedure and may be expected to be similar to other procedures depending on external filtration like trabeculectomy. There is no published data yet about the device.

## 3. Ab Externo Procedures

Ab externo procedures are characterized by using external approach to reach the surgical site, namely, SC or suprachoroidal space, either to remove or modify tissue or to implant a device.

### 3.1. Canaloplasty

**Figure 7:**
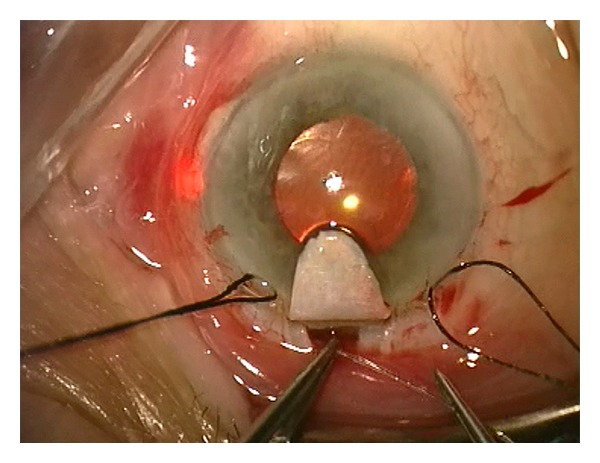
A flexible microcatheter is inserted into Schlemm's canal which is circumferentially viscodilated. The blinking light at the distal tip of the microcatheter helps to identify the track of the microcatheter.

**Figure 8:**
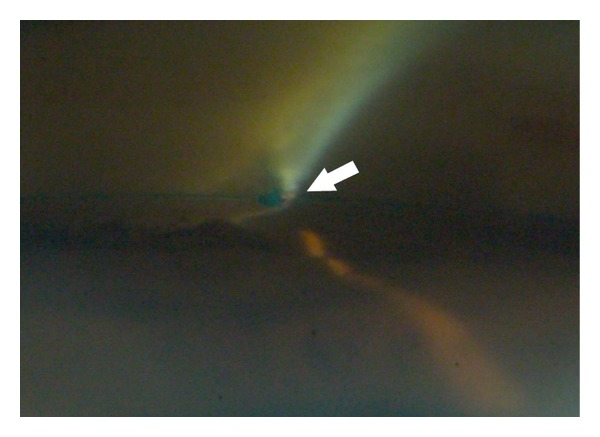
Gonioscopic view of the chamber angle with the suture stent pulling the inner wall of SC towards the anterior chamber.

Canaloplasty is a nonpenetrating and bleb-independent procedure which combines 360 degree viscocanalostomy with a circumferential distention of the canal [24]. The aim of canaloplasty is to restore the physiologic drainage system of eye. Like in viscocanalostomy, parabolic superficial and deep scleral flaps are formed. The deep flap is dissected to the plane of SC which is unroofed. A flexible microcatheter (iTrack-250A, iScience Interventional, Menlo Park, CA, USA) is inserted into SC and advanced 360 degree to dilate stepwise the lumen by injecting microvolumes of sodium hyaluronidate 1.4% (Healon GV, Abbott Medical Optics, Inc., Illinois, USA). The microcatheter has a 200 *μ*m diameter shaft and incorporates an optical fiber to provide an illuminated beacon tip to assist in guidance. The illuminated tip is observed through the sclera during the catheterization at all times to identify the location of the distal tip of the catheter in the canal (Figure 7). Following viscodilation of the full length of canal, a 10-0 polypropylene suture (Prolene, Ethicon Inc.) is sutured to the distal tip of the microcatheter and looped through the canal. The suture is tightened to an extent that it stretches the SC and TM circumferentially (Figure 8). There is wide evidence that canaloplasty lowers IOP to lower-to-mid-teens alone or with combined phacoemulsification (Tables 7 and 8).

An international multicenter prospective study assessed the safety and efficacy of canaloplasty for open-angle glaucoma [24]. Mean baseline IOP was 23.2 mmHg, and mean postoperative IOP was 15.3 mmHg at 1 year, 16.3 mmHg at 2 years, and 15.2 mmHg at 3 years [24, 25]. Mean medication use dropped from 1.8 to 0.6 per patient at 1 year, to 0.6 at 2 years, and to 0.8 at 3 years. A long-term prospective study on canaloplasty included 60 eyes of 60 African patients with a mean follow-up of 30.6 months [29]. The mean preoperative IOP was 45.0 mmHg. Complete success rate (IOP < 21 mmHg without medication) was 77.5%, and qualified success rate (IOP < 21 mm Hg with or without medication) was 81.6% at 36 months. Further, postoperative IOP of ≤21 mmHg did not depend on preoperative IOP. In a randomized controlled trial comparing two tensioning suture sizes, Grieshaber et al. found that IOP reduction was substantial in canaloplasty and slightly greater in combination with 10-0 Prolene than 6-0 Prolene sutures at an equally low complication rate [28]. The mean postoperative IOP without medications was 19.2 mmHg in the 6-0 Prolene group and 16.4 mmHg in the 10-0 Prolene group at 15 months (*P* = 0.04). Further, younger age but not the level of IOP at surgery had a positive effect on the amount of IOP reduction, suggesting that early surgical intervention to reestablish physiological outflow offers the best prognosis. Another prospective study evaluated 32 Caucasian eyes that did not have any surgery prior to canaloplasty. The mean postoperative IOP (without medications) was 12.8 ± 1.5 mmHg at 12 months. The complete success rate for an IOP <21, <18, and <16 mmHg was 93.8% (95% CI 0.86–1.0), 84.4% (95% CI 0.73–0.98), and 74.9% (95% CI 0.61–0.92), respectively [30]. Similarly, a mean postoperative IOP of 12.6 mmHg at 1 year has been recently reported by another study group [32].

An overview of the published complications of canaloplasty is listed in Table 9. The most common adverse event is microhyphema on the first postoperative day. Microhyphema is the result of blood reflux after surgery, indicating a patent or re-established distal outflow pathway and a permeable TM. According to a study of Grieshaber et al., day-one hyphema may be good prognostic sign for IOP reduction [48]. Bleb- or hypotony- related complications are very rare or have not been reported.

The same multicenter study group evaluated the safety and efficacy of combined canaloplasty and cataract surgery in patients with OAG [38]. 54 eyes with a completed one year follow-up were analyzed. The mean baseline IOP was 24.4 mmHg ± 6.1 (SD) with a mean of 1.5 ± 1.0 medications per eye. The mean postoperative IOP was 13.6 ± 3.8 mmHg at 1 month, 14.2 ± 3.6 mmHg at 3 months, 13.0 ± 2.9 mmHg at 6 months, and 13.7 ± 4.4 mmHg at 12 months. Medication use dropped to a mean of 0.2 ± 0.4 per patient at 12 months. Successful circumferential catheterization of the canal was achieved in 44 eyes (81%), and tension sutures were successfully placed in 40 eyes (74%). Surgical complications were reported in 5 eyes (9.3%) and included hyphema (*n* = 3, 5.6%), Descemet tear (*n* = 1, 1.9%), and iris prolapse (*n* = 1, 1.9%). Transient IOP elevation of more than 30 mmHg was observed in 4 eyes (7.3%) 1 day postoperatively. No case of suture erosion through TM or sclera was noted during the follow-up. The authors concluded that canaloplasty combined with phacoemulsification and posterior chamber intraocular lens (IOL) implantation was a safe and effective procedure to reduce IOP in patients with OAG.

### 3.2. Stegmann Canal Expander (SCE)

**Figure 9:**
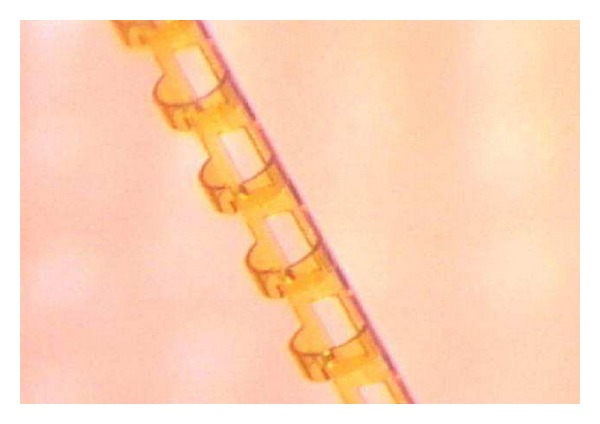
Stegmann Canal Expander design.

**Figure 10:**
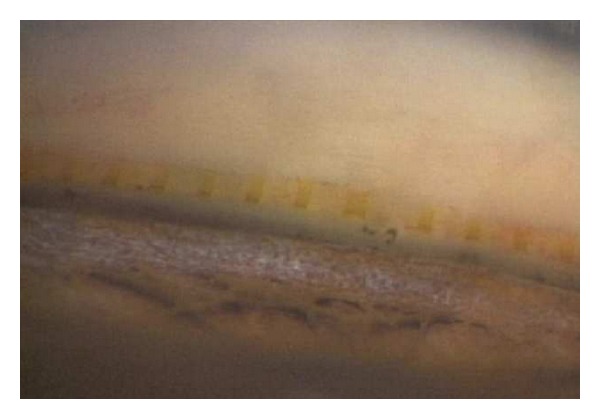
Stegmann Canal Expander in situ (gonioscopic view).

The Stegmann Canal Expander (SCE) (Ophthalmos GmbH, Schaffhausen, Switzerland) is an implant that is made of polyimide and placed into SC to create a permanent distension of the TM. Due to its fenestrations, SCE is patent to aqueous humor (Figure 9). SCE has been developed to make canaloplasty an easier and more reproducible procedure by replacing the suture stent, as proper suture tension is technically very challenging, cannot be measured, and has an inherent risk of cheese-wiring [29]. SCE is implanted as follows: SC is deroofed by preparing a superficial and a deep scleral flap, creating a Descemet window like in viscocanalostomy and canaloplasty. After dilation of the surgical ostia of SC, the microcatheter is inserted in the canal to dilate it circumferentially with highly viscous sodium hyaluronate as described above for canaloplasty. After completed dilation, the catheter is withdrawn, and the SCE implant is placed inside both ostia of SC in order to create a permanent distension of the TM (Figure 10). The rationale behind SCE is to maintain increased permeability of the TM, resulting in increased drainage of aqueous humour from the anterior chamber into SC. The superficial scleral flap is sutured watertight as in canaloplasty to prevent bleb formation and to force the aqueous humour leaving through the physiological drainage system. The device has received the CE market approval in April 2013. Clinical trials are ongoing, but no results have been published to date.

### 3.3. Gold Microshunt

The nonvalved flat plate drainage device made of 24-karat medical-grade gold is 3.2 mm wide, 5.2 mm long, and 44 *μ*m thick (GMS, SOLX, Boston, Massachusetts, USA). It includes several channels for aqueous to percolate which can be opened more with laser energy after surgery, if a further IOP decrease is necessary. The surgeon positions the device through a fornix-based conjunctival flap and under a 4 mm full-thickness scleral dissection into the created suprachoroidal space.

In 2009, a pilot study including 38 patients reported a decrease in IOP from 27.6 ± 4.7 mmHg to 18.2 ± 4.6 mmHg at 11.7 ± 1.3 month [49]. The number of antiglaucomatous medications decreased only from 2.0 ± 0.8 preoperatively to 1.5 ± 1.0 at last visit, meaning that a high percentage of patients needed medications for adequate IOP control despite a distinctive reduction in IOP.

In a prospective uncontrolled case series study with 55 eyes, Figus et al. [50] reported a drop from mean baseline IOP of 27.6 ± 6.9 mmHg to 13.7 ± 2.98 mmHg at 2 year follow-up. Patients included had a diagnosis of refractory glaucoma, had already undergone previous glaucoma surgical intervention, and were pseudophakic, phakic, or aphakic. 67.3% (37 eyes) of the patients achieved qualified success (IOP ≤ 21 mmHg plus a 33% reduction of the presurgical IOP with or without use of medication), while only 5.5% (3 eyes) had complete success (IOP ≤ 21 mmHg without medication). Mild to moderate hyphema was the most reported complication. Bullous choroidal detachment, corneal edema, and exudative retinal detachment were also described, which sometimes lead to shunt removal. The biggest problems of any devices which are inserted into the suprachoroidal space are the high risk of fibrosis and inherent failure of the procedure as confirmed by Agnifili et al. [51] demonstrating fibrosis inside the shunt grid or encapsulating the device.

At present, two ongoing studies are listed with the device, including refractory glaucoma patients. The disruption of conjunctival integrity is not an advantage, but the risk of hypotony would be avoided creating a new outflow pathway without filtering bleb. The safety and efficacy of such permanent implant communicating the anterior chamber and the suprachoroidal space need to be investigated in further studies.

## 4. Comparison of MIGS to Trabeculectomy

Currently, there are only a few reports comparing MIGS to fistulating trabeculectomy.

In a retrospective study [35], canaloplasty was compared with trabeculectomy regarding safety and efficacy. The IOP dropped from 21.2 ± 6.6 mmHg preoperatively to 13.8 ± 4.9 mmHg (32% reduction of IOP) in the canaloplasty group and from 23.4 ± 10.4 mmHg to 11.6 ± 4.0 mmHg (43% reduction of IOP) in the trabeculectomy group at 12 months (*P* = 0.03). Also, the study confirmed the different profile of complications between the two techniques. Among canaloplasty patients, the most common complications were hyphema (21%) followed by peripheral anterior synechiae (6%) and Descemet's detachment (3%). In the trabeculectomy group, choroidal detachment was observed in 17% of the patients, followed by bleb revision (15%), hypotony maculopathy (4%), and suprachoroidal hemorrhage (2%). Brüggemann et al. compared trabeculectomy and canaloplasty between both eyes of the same patient [37]. In this consecutive case series of 30 eyes, mean IOP reduction was of 50.3% (26.73 ± 6.4 mmHg preoperatively to 13.21 ± 2.83 mmHg postoperatively) in canaloplasty, while IOP decreased by 53.4% in trabeculectomy (26.3 ± 10.9 mmHg preoperatively to 15.2 ± 11.2 mmHg postoperatively). In the canaloplasty group, only two eyes required further intervention (anterior chamber reformation and goniopuncture), while in the trabeculectomy group, a total of 112 procedures were necessary to control IOP (5-FU injection, 5-FU needling, bevacizumab injection, laser suture lysis, anterior chamber reformation, bleb revision, cataract surgery, and conjunctival closure). Furthermore, it is important to emphasize that canaloplasty patients needed an average of 3.9 ± 0.8 follow-up visits, while trabeculectomy patients needed 8.5 ± 3.6 follow-up visits, indicating that bleb-independent procedures need less postoperative care.

One recent study evaluated the results after combined procedures—phacotrabeculectomy and phacocanaloplasty [52]. In the phacotrabeculectomy group, IOP decreased from 30.0 ± 5.3 mmHg to 11.7 ± 3.5 mmHg, while in phacocanaloplasty group, the observed decrease was from 28.3 ± 4.1 mmHg preoperatively to 12.6 ± 2.1 mmHg postoperatively. Though phacotrabeculectomy group showed greater IOP decrease results, there was no statistical significance over time between the two groups. The incidence of complications had no statistical significance between groups, but phacotrabeculectomy-related complications showed a greater risk profile like choroidal detachment, bleb leakage, and the need for anterior vitrectomy. Furthermore, the number of invasive postsurgical interventions like laser suture lysis and subconjunctival injections (needling) was greater in the phacocanaloplasty group.

Besides comparison regarding safety and efficacy, any new surgical techniques and devices must have a good cost-value ratio for the health care system in order to become a true alternative to classic fistulating surgery. Currently, there is only one study comparing the costs between a new procedure, that is, canaloplasty and trabeculectomy [53]. The mean duration of hospitalization was 5.3 ± 0.8 days in the canaloplasty group, whereas it was 10.7 ± 2.8 days in the trabeculectomy group. In the beginning, operating time was greater in canaloplasty which directly implies greater costs; however, with larger surgeon's experience, there was a great decrease in surgical time. Although both procedures achieved good IOP control, longer hospitalization, higher readmission rates, and more postoperative visits in the trabeculectomy group resulted in a higher total cost of 1658,50 € per patient compared to 821,50 € per patient in the canaloplasty. This comparison may be exemplarily for other MIGS procedures. One must not only consider the expenses for the new device itself, but also the time and effort for the follow-up visits, including postoperative interventions which ultimately reflect better quality of life for the patient and health professionals and lower health care costs.

## 5. Conclusion

In the past few years, glaucoma surgeons experienced a significant increase in the offered numbers of new glaucoma devices and surgical technology. The common goal of MIGS is to lower IOP as equal as possible to established procedures (i.e., trabeculectomy or tube implant), but avoiding most, if not all, of the serious and potentially devastating complications of fistulating procedures. In addition, most of the MIGS procedures are fast to perform and have a more rapid visual recovery than fistulating surgery. They claim to have a low complication rate, are antimetabolite free, and maintain the conjunctiva integrity in case of ab interno approach. For these reasons, MIGS is suitable for combined cataract surgery.

Current published data of MIGS show that IOP levels after surgery are in the mid- to higher teens; thus, MIGS is often used to postpone a more invasive surgical intervention in the early to moderate stage of glaucoma, to prolong patient's adhesion to treatment, and to improve the quality of life. Further, there is limited data on costs comparing MIGS to trabeculectomy. However, it is likely that the MIGS may not be more expensive than trabeculectomy if the postoperative visits and interventions are taken into account.

Trabectome and canaloplasty achieve on average a lower IOP than the trabecular microbypass, possibly because they are targeting a larger area of diseased TM. Understanding the different principles of the new devices and techniques, proper surgical training, and careful patient selection are important requirements for a successful implementation of MIGS. However, as there is limited clinical experience and evidence with many of the new devices whereof some are still under investigation today, more prospective studies are needed before MIGS can be fully recommended for a widespread use in the near future.

## Figures and Tables

**Table 1:** Overview of current glaucoma procedures with MIGS.

Surgical Approach	Internal Filtration	External Filtration
Ab Interno	Trabeculotomy (Trabectome, Excimer Laser) Trabecular micro-bypass (iStent), Suprachoroidal stent (Cypass) Intracanalicular scaffold (Hydrus)	Subconjunctival implant (Aquesys)
Ab Externo	Canaloplasty Stegmann Canal Expander Suprachoroidal Gold micro shunt	

**Table 2:** Frequency of reported Trabectome complications.

Type	Frequency in %
Hyphema	0–59
Goniosynechiae	0–14
Corneal epithelial defects	0–3
IOP spike	0–5
Iris and lens touch	0–1.3
Infection	0
Bleb formation	0
Wound leaks	0
Choroidal effusion	0
Choroidal hemorrhage	0

**Table 3:** Trabectome studies results.

Author	Year	Number of eyes	Followup (months)	IOP reduction (mmHg)	IOP reduction (%)	Postop. IOP (mmHg)	Reduction in meds (%)
Trabectome alone							
Minckler et al. [2]	2005	37	12	11.3 ± 1.4	40%	16.3 ± 1.3	0.8
Minckler et al. [3]	2006	101	30	11.3 ± 2.9	40%	16.3 ± 3.3	N/A
Vold [4]	2011	897	6	1.1–17.7	6–48%	14.2–17.9	25–35%
Mosaed et al. [5]	2010	538	12	9.3 ± 3.3	31%	16.6 ± 4.4	0.8 (28%)
Trabectome/Phaco							
Francis et al. [6]	2008	304	21	3.3 ± 2.8	25	16.7 ± 3.5	1.2 ± 0.1
Francis [7]	2010	114	12	6.8 ± 2.8	28%	15.3 ± 3.5	40%
Vold [4]	2011	504	6	1.1–16.2	7–47%	13.5–17.8	28–33%
Mosaed et al. [5]	2010	290	12	4.6 ± 3.2	18%	15.6 ± 3.7	0.75 (33%)
Francis et al. [8]	2011	112	12	6.7 ± 2.4	N/A	15.4 ± 3.1	N/A
Maeda et al. [9]	2013	80	6	9.2 ± 2.8	29%	17.4 ± 3.4	2.3.2 ± 1.2

**Table 4:** ELT study results.

Author	Year	Number of eyes	Followup (months)	IOP reduction (mmHg)	IOP reduction (%)	Postop. IOP (mmHg)	Reduction in meds
ELT only							
Babighian et al. [10]	2006	21	24	7.8 ± 0.07	31.8	16.9 ± 2.1	0.71 ± 0.8
Babighian et al. [11]	2010	15	24	7.4	29.6	19.1 ± 1.8	0.73 ± 0.8
ELT/Phaco							
Wilmsmeyer et al. [12]	2006	60	12	10.7 ± 1.7	47	12.8 ± 1.5	1.8 ± 0.9
Töteberg-Harms et al. [13]	2011	24	12	8.9 ± 5.2	34.7	16.5 ± 4.9	0.79 ± 0.6

**Table 5:** Microbypass stent studies results.

Author	Year	Number of eyes	Followup (months)	IOP reduction (mmHg)	IOP reduction (%)	Postop. IOP (mmHg)	Reduction in meds (%)
iStent alone							
Spiegel et al. [14]	2007	6	12	4.9 ± 2.6	24.2	15.3 ± 3.7	0.5 ± 0.3
Buchacra et al. [15]	2011	10	12	6.6 ± 5.4	27.3	19.9 ± 2.3	1.8 ± 0.1
iStent/Phaco							
Spiegel et al. [16]	2008	48	6	5.7 ± 3.8	26.5	15.8 ± 3.0	1.5 ± 0.7
Spiegel et al. [17]	2009	42	12	4.4 ± 4.5	21.3	16.9	1.2 ± 0.7
Fernández-Barrientos et al. [18]	2010	17	12	6.57 ± 2.95	27.2	17.6 ± 2.8	1.12 ± 0.48
Fea [19]	2010	12	15	3.2 ± 3.0	17.3	14.8 ± 1.2	2.0 ± 0.9
Samuelson et al. [20]	2011	106	12	8.4 ± 3.6	31	17	1.4 ± 0.8
Arriola-Villalobos et al. [21]	2012	19	54	3.16 ± 3.9	16.3	16.3 ± 4.2	0.47 ± 0.96
Craven et al. [22]	2012	240	24	—	8.4	17.1 ± 2.9	0.3
Belovay et al. [23]	2012	53	24	3.16	—	14.3 ± 2.9	2.0 ± 1.4

**Table 6:** Frequency of microbypass stent complications.

Type	Frequency in %
Mild hyphema	0–70
Transient IOP spike	0–30
Corneal edema	0–20
Transient IOP spike	0–21
Stent obstruction	4–14.9
AC collapse	0–2.3
Inability to implant stent	0–2.3
Vitreous incarceration	0–2.3
Stent malposition	0–21.4
Secondary surgery	0–4.5

**Table 7:** Canaloplasty alone.

Author	Year	Number of eyes	Followup (months)	IOP reduction (mmHg)	IOP reduction (%)	Postop. IOP (mmHg)	Meds reduction (%)
Lewis et al. [24]	2007	74	12	8.6 ± 0.5	35.8	16.2 ± 3.5	1.3 (68)
Lewis et al. [25]	2009	84	24	6.9 ± 0.1	29.3	16.3 ± 3.7	1.4 (70)
Lewis et al. [26]	2011	103	36	8.6 ± 1.5	34	15.5 ± 3.5	1.0 ± 0.1
Peckar and Koerber [27]	2008	97	18	13.1 ± 5.2	48	14.1 ± 3.2	2.4 (76)
Grieshaber et al. [28]	2010	90	15	28.6 ± 7.2	36.4	16.2 ± 4.9	—
Grieshaber et al. [29]	2010	60	36	31.7 ± 6.7	65.8	13.3 ± 1.7	—
Grieshaber et al. [30]	2011	32	18	14.2 ± 2.1	47.2	13.1 ± 1.2	2.6
Koerber [31]	2012	15	18	12.0 ± 0.1	45.3	14.5 ± 2.6	1.7 (85)
Matthaei et al. [32]	2011	46	12	5.6 ± 3.2	30.7	12.6 ± 2.4	1.3 (43)
Bull et al. [33]	2011	82	36	7.9 ± 1.3	34.3	15.1 ± 3.1	0.9 (53)
Fujita et al. [34]	2011	11	12	8.4 ± 1.8	35.9	15.0 ± 4.1	1.6 (25)
Ayyala et al. [35]	2011	33	12	7.4 ± 1.5	32	13.8 ± 4.9	2
Klink et al. [36]	2012	20	9	10.6 ± 4.2	32.5	13.3 ± 9.9	2.6 (82)
Brüggemann et al. [37]	2013	30	12	14.6 ± 4.5	50.3	13.2 ± 2.8	2.5 (100)

**Table 8:** Combined canaloplasty with phacoemulsification.

Author	Year	Number of eyes	Followup (months)	IOP reduction (mmHg)	IOP reduction (%)	Postop. IOP (mmHg)	Meds reduction (%)
Lewis et al. [24]	2007	13	12	10.7 ± 1.8	45.5	12.8 ± .3.6	NA
Shingleton et al. [38]	2008	54	12	10.7 ± 1.7	43.8	13.7 ± 4.4	1.3 (86)
Lewis et al. [26]	2011	54	36	9.8 ± 2.6	42.1	13.6 ± 3.6	1.0 ± 0.1
Bull et al. [33]	2011	16	36	10.5 ± 2.8	43.2	13.8 ± 3.2	1.0 (66)

**Table 9:** Intra- and postoperative complications of canaloplasty.

Type	Frequency in %
Gross hyphema	1.6–6.1
Descemet's detachment	1.6–6.1
360° cannulation impossible	0–9
False passage	3.3–12.1
IOP spike > 30 mm Hg	1.6–8.7
Cataract formation	0–8.4
Suture cheese wiring	0–1.6
Flat anterior chamber	0–2.2
Persistent hypotony	0–0.8
Choroidal detachment	0
Bleb formation	0–3.8
Blebitis, endophthalmitis	0
